# Stem Cell-Derived Exosomes and Nanovesicles: Promotion of Cell Proliferation, Migration, and Anti-Senescence for Treatment of Wound Damage and Skin Ageing

**DOI:** 10.3390/pharmaceutics12121135

**Published:** 2020-11-24

**Authors:** Hyeonjin Cha, Seyoung Hong, Ju Hyun Park, Hee Ho Park

**Affiliations:** 1 Department of Medical Biomaterials Engineering, Kangwon National University, Chuncheon-si 24341, Gangwon-do, Korea; hyeon_j03@kangwon.ac.kr; 2 Interdisciplinary Program in Biohealth-Machinery Convergence Engineering, Kangwon National University, Chuncheon-si 24341, Gangwon-do, Korea; syh@kangwon.ac.kr; 3 Department of Biotechnology and Bioengineering, Kangwon National University, Chuncheon-si 24341, Gangwon-do, Korea

**Keywords:** stem cell, exosome, cell-engineered nanovesicle (CNV), proliferation, migration, anti-senescence, wound healing, anti-ageing

## Abstract

Extracellular vesicles (EVs), such as exosomes, are nano-sized vesicles derived from endocytic membranes and contain biomolecules such as proteins, lipids, RNAs, and DNAs for the transfer of signals to recipient cells, playing significant roles in cell-to-cell communication. Discovery of exosomes has attracted attention for possible use as next generation therapies in clinical applications; however, several studies suggest that cells secrete exosomes that perform as mediators in the tumor niche and play several roles in tumorigenesis, angiogenesis, and metastasis. Recently, stem cell-derived exosomes have been suggested as a desirable source for regenerative medicine due to their roles in the promotion of angiogenesis via migratory and proliferative mechanisms. This review is aimed at demonstrating the present knowledge of stem cell-derived exosomes and cell-engineered nanovesicles (CNVs) as proliferative, migratory, and anti-senescent therapeutic biomaterial for use in tissue regeneration; wound healing and anti-ageing are explained. We conclude this review by discussing the future perspectives of stem cell-derived exosomes and CNVs as a platform in therapeutic strategies for treatment of wound damage and skin aging.

## 1. Introduction

Communication between cells is paramount in order to maintain homeostasis and cellular functions in multicellular organisms. The intercellular communication is mediated in several ways, including direct cell–cell interaction and transfer biomolecules. For communication via transfer of biomolecules, cells release extracellular vesicles (EVs), such as exosomes, microvesicles (MVs), and apoptotic bodies derived from endosomes, plasma membrane, and cell lysis, respectively [1]. These EVs contain cytosolic proteins, lipids, RNAs, and DNAs and act as carriers to recipient cells. The contents indicate the metabolic status of the donor cells, and therefore contents of EVs transferred to target cells play a vital role in intercellular communication. It is considered that exosomes are not only involved in cell-to-cell communication but also in the progression and metastasis of several diseases such as cancer [2,3].

**Figure 1:**
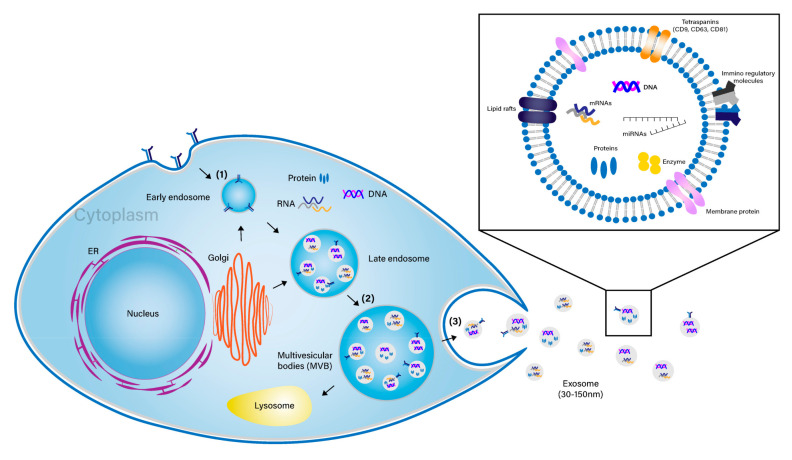
Production of small nano-sized membrane lipid vesicles termed “exosomes”. Exosomes are secreted by most cell types with a diameter ranging from 30–150 nm. Exosome biogenesis occurs via a few steps: (1) early endosomes undergo a series of maturation steps forming late endosomes, named multivesicular bodies (MVBs), (2) fusion of endosome-derived MVBs with the cell membrane, and (3) release of exosomes into the extracellular environment through exocytosis mechanism.

EVs can be divided into three classes depending on their mechanism of release and size: (1) “apoptotic bodies” that are bigger than 1000 nm, (2) “MVs” that are 100–1000 nm, and (3) “exosomes” that are less than 150 nm. Exosomes are small nano-sized membrane lipid vesicles that are secreted by most cell types with a diameter ranging from 30–150 nm (Figure 1). Exosome biogenesis occurs via a few steps: (1) early endosomes undergo a series of maturation steps forming late endosomes, named multivesicular bodies (MVBs), (2) fusion of endosome-derived MVBs with the cell membrane, and (3) release of exosomes into the extracellular environment through the exocytosis mechanism [1,4,5,6,7]. Exosomes contain not only numerous proteins, including intracellular enzymes, surface receptors, transcription factors, and cytokines, but also nucleic acids such as DNA, mRNA, miRNA, and other non-coding RNA, which can modulate the physiologies of the recipient cells [8]. The information for exosome contents can be obtained from web-based databases, such as Vesiclepedia, Exocarta, and EVpedia. A recent study has analyzed the data provided on the Vesiclepedia website (http://www.microvesicle.org) and reported that 32,267 unique proteins, 17,005 unique mRNAs, and 2431 unique miRNAs have been identified from 1254 EV studies [9]. Other than the conventional biogenesis approach of generating exosomes, a novel bioinspired approach has also been used to generate exosome-mimetic nanovesicles that are less than 150 nm, called cell-engineered nanovesicles (CNVs), by mechanical disruption of plasma membranes [10].

The specificity and binding of released exosomes to the recipient cells is controlled by adhesion molecules, such as integrin on the surface of cell membranes. Exosomes are involved in various cellular functions, biological processes, and pathological conditions, such as angiogenesis, inflammation, cancer, immune response, cell death, and neurodegeneration [11]. Exosomes are generated from various types of cells, including fibroblasts, adipocytes, intestinal epithelial cells, neurons, cancer, and tumor cells. The released exosomes are found in many biological fluids, such as blood, saliva, urine, breast milk, amniotic fluid, malignant effusions, and ascites.

Until recently, it has been reported that stem cells secrete various growth factors that promote tissue regeneration, such as basic fibroblast growth factor (bFGF), vascular endothelial growth factor (VEGF), and transforming growth factor-β (TGF-β), which is a characteristic of stem cells as distinguished from other cell types [12]. Although inducing the paracrine actions of the transplanted stem cells has been used to regenerate injured tissues, some drawbacks were also revealed, including low regeneration efficiency due to poor cell viability and engraftment [13,14]. Unexpected alteration in the physiologies of the transplanted stem cells, such as tumorigenesis, is another limitation of direct stem cell therapy. For the reason, many studies have focused on exosome among several stem cell-derived paracrine factors as “cell-free” therapeutic agent [15]. Exosome contents depend on the type of the donor cell and reflect the cellular characteristics of the donor cell, which indicate that exosomes can deliver several therapeutic factors derived from various types of stem cells. Compared to the single cytokine therapy, exosomes that contain a wider variety of active factors can lead to more significant therapeutic effects. Although it is difficult to chemically define all components contained within, exosomes preserve the biological activities of labile cytokines during in vivo applications and facilitate efficient delivery of active factors across several tissues due to their cell-derived lipid bilayers [16,17]. Several studies have shown that exosomes derived from stem or progenitor cells can be used to improve cellular functions and phenotypic symptoms, and treat various injuries, such as ischemia/reperfusion injury [18,19], myocardial infarction [20,21], cutaneous closure [7,22,23], renal, and lung injuries [24,25].

Recently, studies have shown that stem cell-derived exosome play significant roles in proliferation, migration and anti-senescence for possible use in tissue regeneration; wound healing and anti-ageing. This review demonstrates the current stage and advances of our knowledge in the biological functions, significance, and role of stem cell-derived exosomes and CNVs for use in treatment of cell regeneration. In particular, exosomes and cell-engineered nanovesicles (CNVs) are categorized according to cell types and therapeutic applications with respect to tissue regeneration, such as wound healing and anti-ageing are explained in detail.

## 2. Somatic and Cancer Cell-Derived Exosomes

In previous studies, exosomes derived from platelets have demonstrated an increase in proliferation, expression of angiogenic factors, invasion, and promotion of tumor chemotaxis and metastasis [26,27]. Exosomes have also been associated with the proliferation and lifespan of fibroblasts [28]. Previously, it was shown that exosomes derived from senescent fibroblasts can promote cell proliferation of MCF-7 human breast cancer cells [29]. In addition, it was reported that vasorin delivery using exosomes promoted migration of human umbilical vein endothelial cells through receptor-mediated endocytosis of exosomes [30]. Moreover, exosomes released from progenitor cells, such as cardiomyocyte progenitor cells, have been shown to stimulate the migration of endothelial cells [31]. However, a previous experiment showed that exosomes could be used as RNAi vectors in gene therapy. It was demonstrated that exosomes derived from bone marrow stromal cells (transfected with miRNA expression plasmid) can downregulate the endothelial growth factor (EGF) receptor and inhibit the proliferation of glioma cells in vivo [32]. In addition, when exosomes were loaded with paclitaxel, doxorubicin, or Withaferin A against A549 cells, the chemotherapeutic agents were effectively released and suppressed the proliferation lung cancer cells in vitro and in vivo [33]. Similarly, when nanovesicles were loaded with chemotherapeutic drugs, increase in TNF-α-stimulated endothelial cell death in vitro and trafficking to tumor tissue were observed [10]. Subsequently, a reduction in tumor growth without the adverse effects in vivo followed.

Exosomes derived from other types of cells, such as exosomes with CD147 (also known as basigin or extracellular matrix metalloproteinase inducer) from trophoblast cells have been shown to regulate growth, angiogenesis, and tissue remodeling in the placenta [34,35]. Pretreating exosomes to human umbilical vein endothelial cells (HUVECs) and subcutaneous injection in nude mice showed an increase in the neovascularization in the peri infarct area of the heart and angiogenesis in vivo [36]. Exosomes released from progenitor cells stimulated endothelial cell proliferation [37], cell migration [31], tissue vascularization, and angiogenesis [38].

Proliferation and migration of cancer cells is an important factor in cancer metastasis. It has been reported that exosomes derived from a tumor can induce the proliferation of tumor cells. It was shown that exosomes released from glioblastoma induced proliferation of human glioma cell line U87 [39]. Normally, tumor-derived microvesicles, such as exosomes, serve as a means of delivering proteins and genetic information to recipient cells in the tumor environment. The tumor-specific epidermal growth factor receptor variant III (EGFRvIII) was detected in serum exosomes from 7 out of 25 glioblastoma patients, demonstrating that tumor-derived exosomes may provide diagnostic information through a blood test. In addition, exosomes released from T24 tumor cells induced proliferation of human urinary bladder carcinoma cell lines T24 and 5637 via inhibition of tumor cell apoptosis and activation of the Akt and extracellular signal-regulated kinase (ERK) pathways [40]. Further analysis showed upregulation of Bcl-2 and Cyclin D1 protein expression levels, but reduction of Bax and caspase-3 proteins levels, indicating that cancer cell-derived exosomes have a potential to inhibit tumor cell apoptosis via activation of Akt and ERK pathway genes. Similarly, it was shown that exosomes released from murine melanoma promoted growth of murine melanomas in vivo [41]. It was shown that systemic treatment of melanoma-derived exosomes inhibited apoptosis and accelerated the growth of melanoma tumors in mice. On the other hand, when exosomes released from bladder cancer cells were treated to other cells, they were internalized and promoted proliferation and expression of cancer-associated markers [42]. Healthy primary bladder fibroblasts (HFs) were induced into cancer-associated fibroblasts (CAFs) and expressed CAF markers by bladder cancer-derived exosomes, demonstrating that exosomes derived from cancer cells promote aggressive phenotypes of non-invasive cells. Likewise, exosomes released from CAFs have shown evidence that support tumor growth by providing nutrients to malignant cells [43]. Moreover, exosomes released from prostate cancer cells in hypoxic condition increased the motility and invasiveness of naïve prostate cancer cells by inducing microenviroment changes [44]. These results collectively indicate that exosomes can promote aggressiveness of the tumor cells. In another study, it was demonstrated that exosomes derived from CAFs can regulate proliferation and survival of pancreatic cancer cells [45]. Exosomes released from colorectal cancer cells in hypoxic condition promoted proliferation and beta-catenin nuclear translocation of endothelial cells [46]. Exosomes from gastric cancer cells have also been shown to promote direct and indirect proliferation of tumor cell proliferation via PI3K/Akt and mitogen-activated protein kinase (MAPK)/ERK activation [47]. However, in a previous study it was shown that exosomes released from human prostate cancer cells can induce T-cell apoptosis through the FasL pathway and inhibit T-cell proliferation [48]. In an another report, exosomes released from gastric cancer cells induced nuclear factor kappa-light-chain-enhancer of activated B cells (NF-κB) activation in macrophages and led to secretion of pro-inflammatory cytokines, such as tumor necrosis factor alpha (TNF-)α, interleukin 6 (IL-6), granulocyte colony-stimulating factor (G-CSF), and C-C motif chemokine ligand 2 CCL-2, which in turn increased proliferation and migration of gastric cancer cells [49]. Similarly, exosomes released from lymphoma cell lines induced proliferation and differentiation of B cells [50]. However, miRNA containing exosomes have shown the opposite effects. miR-302b loaded exosomes suppressed proliferation and migration of lung cancer cells via inhibition of TGF-βRII [51]. Synthetic miR-143 loaded exosomes have been shown to inhibit migration of osteosarcoma cells [52].

It has been reported that exosomes play a substantial role in the invasion in tumor-associated angiogenesis and tumor growth. Angiogenesis is the term used to describe the formation of newly generated capillaries from existing blood vessels and it is mediated by a process involving the migration, growth, and differentiation of endothelial cells [53,54]. Exosomes derived from various cells including cancers and tumors play an important role in angiogenesis [55]. It was shown that exosomes derived from melanoma cells can recruit the spread of melanoma and stimulate factors associated with metastasis, such as extracellular matrix remodeling and angiogenesis [56]. Exosomes derived from cancer cells are taken up by endothelial cells and stimulate secretion of pro-angiogenic materials and results in angiogenesis under hypoxic conditions [57]. It is proposed that cancer cell-derived exosomes enhance the vascular permeability and metastatic spreading by downregulating the structural integrity protein, ZO-1, and thus weakening the tight junction between endothelial cells [58]. Interestingly, it was shown that cancer-derived exosomes can transform stem cells into cancer stem cells, where prostate cancer cell-derived exosomes were able to transform local prostate tissue stem cells into cancer stem cells (CSCs) [59]. In another report, it was demonstrated that glioma cell-derived exosomes enhanced the rates of proliferation, migration, and invasion of bone marrow mesenchymal stem cells (BM-MSCs) [60]. Moreover, alteration in intracellular protein production, including the production of the metastasis-related proteins were observed, indicating change to a “tumor-like” phenotype in BM-MSCs, demonstrating the possible dangers of using cancer-derived exosomes.

## 3. Cancer Stem Cell-Derived Exosomes

Cancer stem cells (CSCs) are a small subpopulation of cells within tumors, and are known to share similar characteristics to normal progenitor or stem cells. These CSCs have a self-renewal property, differentiation ability to multiple lineages, and tumorigenicity. There is still a debate on the origin of CSCs, whether they are produced from non-stem cells or normal stem cells. Nonetheless, extensive studies and growing evidences suggest that CSCs promote angiogenesis, metastasis, and drug resistance [61]. Interaction of CSCs in local cancer cell environment enable regulation of tumor growth and progression of cancers [62,63]. It was shown that exosomes released from CSCs increase angiogenesis of endothelial cells [64]. In addition, MVs released from human renal cancer stem cells promoted angiogenesis [65]. It is considered that exosomes released from the CSCs stimulate angiogenesis by increasing the expression of vascular endothelial growth factor receptor 1 (VEGFR1) in endothelial cells [66]. The results demonstrate that exosomes derived from cancer stem cells have therapeutic potential for angiogenesis (Table 1).

In particular, it has been shown that any changes made to the parent cell causes alteration to the content and secretion of exosomes, thereby altering the information [67]. It was noticed that the miRNA contents are different between the exosomes derived from CSCs and exosomes derived from non-stem prostate cancer cells [68], and CSC-derived exosome miRNAs also serve to create a tumor microenvironment by promoting the expression of VEGF [64]. For example, glioblastoma stem cell-derived exosomes have been reported to create an immunosuppressive microenvironment by inducing monocytes towards the M2 macrophage phenotype via signal transduction and activators of the transcription-3 (STAT3) pathway [69]. In another study, exosomes derived from gastric CSCs were characterized and it was shown that the measurement of identified 11 miRNAs in patient serum could be used as an indicator for cancer metastasis [70]. Recent studies have also reported that exosomes derived from CSCs promote metastasis by enhancing epithelial-mesenchymal transition (EMT) through metastasis [71,72]. In another study, it was shown that exosomes derived from colorectal CSCs enhance the production of IL-1β and promote a pro-tumoral phenotype in neutrophils [73]. Furthermore, there is an evidence that EVs derived from CSCs could influence tumor cell fate by genetic reprogramming of resident cells and tumor microenvironment, including immune cells [74]. These findings demonstrate the possible dangers of using CSC-derived exosomes and also suggest that their roles still need to be deeply understood.

## 4. Multipotent Stem Cell-Derived Exosomes

**Figure 2:**
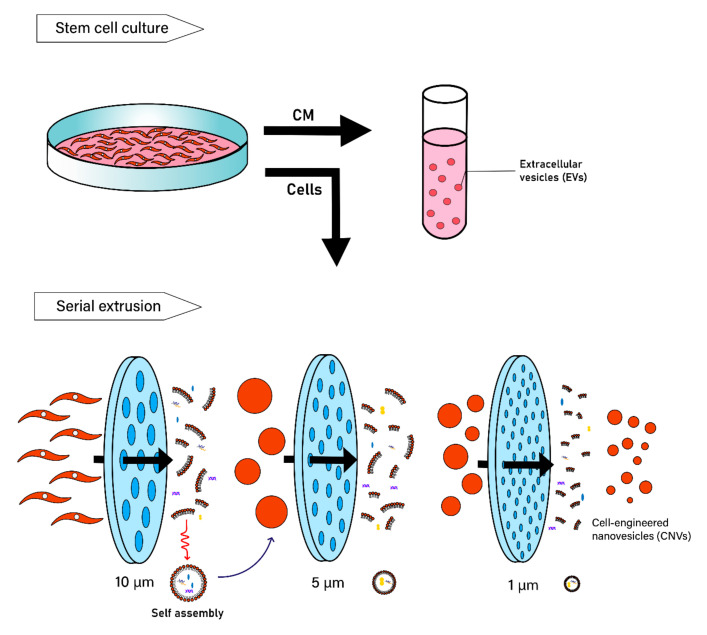
Approaches to generating nano-sized vesicles. Preparation of nano-sized vesicles by collection of stem cell conditioned media containing secreted exosomes or serial extrusion of cells through membrane filters. The latter forced mechanical disruption of plasma membrane method generates extracellular vesicle (EV)-mimetic nanovesicles, termed cell-engineered nanovesicles (CNVs).

In regenerative medicine, stem cells represent a new paradigm in cell therapy because of their self-renewal proliferative property and potency to differentiate into various types of cells. Materials such as exosomes derived from stem cells have been widely used in various clinical applications. It has been reported that exosomes play a pivotal role in communication between cells and play an important role in biological processes, including intercellular communication, development and differentiation of stem cells, and immune function [75]. Stem cells and their paracrine factors, such as TGF-β, platelet-derived growth factor (PDGF), and basic fibroblast growth factor (bFGF) are potential therapeutic drugs for the treatment of diseases as well as tissue regeneration and would healing, and several studies have reported that exosomes are involved in playing an important role in their functions [18,19,76,77]. Several studies have identified numerous proteins, including paracrine factors for treatment of damaged tissues [78] and modulation of angiogenesis [79]. Reports have shown that EVs released from mesenchymal stem cells (MSCs) under stress promote migration and apoptosis resistance of osteosarcomas [80]. It was also shown that exosomes derived from MSCs promote proliferation and migration of both normal and chronic wound fibroblasts, and also increase angiogenesis [81]. Exosomes loaded with miR-132 from MSCs induced angiogenesis by increasing the tube formation of endothelial cells. Exosomes derived from MSCs containing miRNAs, such as miR210, miR126, miR132, and miR21, showed that they play significant roles in angiogenesis [82]. In addition, exosomes derived from MSCs containing miR-191, miR-222, miR-21, let-7a regulated cell cycle progression, proliferation, whereas exosomes released from MSCs containing miR-222, miR-21, let-7f modulated angiogenesis [83]. Due to these promising effects, exosomes released from human umbilical cord MSCs (UC-MSCs) were used to enhance angiogenesis and promote skin repair after second-degree burn injury [84]. Additionally, exosomes from human induced pluripotent stem cell–derived MSCs (iPSC-derived MSCs) showed protection of liver against hepatic ischemia/reperfusion injury [85,86]. Similarly, exosomes derived from human iPSC-derived MSCs showed repair of bone defects through improved angiogenesis and osteogenesis [87]. Although stem cell conditioned media containing exosomes can be used, it was shown that exosomes released from MSCs significantly promoted myogenesis and angiogenesis than MSC-conditioned media [88] (Figure 2, upper panel).

Many studies have explored the beneficial effects of materials released from multipotent stem cells on tissue regeneration and skin aging. Numerous studies have reported that EVs, such as exosomes derived from MSCs, can be used as a potential therapeutic tool for tissue repair and wound regeneration [89,90]. Preclinical and clinical studies have shown that MSCs can be applied for tissue regeneration and wound healing [91,92,93]. Within the lipid bilayer membrane of exosomes and EVs, there are RNAs, proteins, and cytokines which can induce repair post cellular injury [94]. It has been reported that exosomes derived from human iPSC-MSCs enhance collagen synthesis and angiogenesis, thereby promoting cutaneous wound healing [95]. Moreover, it was shown that exosomes derived from human adipose-derived stem cells (ADSCs) accelerate and promote cutaneous wound recovery of fibroblasts [96]. Additional evidences from recent reports support that exosomes secreted from iPSC-derived MSCs accelerate skin cell proliferation and skin would closure [97,98].

Several reports have shown that MSC-derived exosomes have the effect of stimulating the regeneration of damaged peripheral nerves. Bone marrow mesenchymal stem cells (BM-MSCs)-derived exosomes have been shown to improve nerve histomorphometric repair and increase marker expression of axon regeneration [99]. Similarly, EVs from UC-MSCs have shown restoration of peripheral nerves [100]. UC-MSCs-derived EV promoted the production of axons and Schwann cells that regulate inflammation through down-regulation of inflammatory cytokines and up-regulation of anti-inflammatory cytokines. The influence of EVs derived from MSCs was also studied in spinal cord injury (SCI), which is accompanied by damage to the microvascular integrity and increase in the permeability of the blood–spinal cord barrier (BSCB) [101]. BM-MSC-derived EV improves the structural integrity of BSCB and improves motor function in SCI rat models [102]. Other studies have suggested the prevention of neuronal cell apoptosis through activation of the Wnt/β-catenin signaling pathway [103]. In a previous study, it was reported that neural stem cell (NSC)-derived EV activates autophagy after SCI to attenuate apoptosis and nerve inflammation. It is considered that this is associated with autophagy, where NSC-sEV was shown to mediate autophagy-related pathways [104]. It was demonstrated that the expression of the anti-apoptosis protein Bcl-2 is up-regulated and that the inflammatory cytokines, such as IL-1β and IL-6, and TNF-α are reduced in expression. Recently, several advantages of exosomes derived from UC-MSCs and ADSCs were shown when compared to their parental cells. The UC-MSC and ADSC-derived exosomes are drawing more attention as potential new tools for the treatment of tissue injury by means of an additional mode of actions, such as autophagy regulation, downregulation of proinflammatory cytokines, upregulation of anti-inflammatory cytokines, and reduced oxidative stress and damage [105]. In all, the results demonstrate that exosomes derived from multipotent stem cells have therapeutic potential not only for proliferation, migration, and angiogenesis but also for treatment of muscle and nerve injury, ischemia-reperfusion injury, bone defects, wound damage, and apoptosis (Table 2).

## 5. Pluripotent Stem Cell (PSC) Derived Exosomes and CNVs

Exosomes derived from stem cells have been explored for use in therapeutic applications. However, most studies on exosomes have focused on multipotent stem cells, and to date, study of the therapeutic effects of exosomes derived from pluripotent stem cells (PSCs) is limited. In recent years, several reports have demonstrated that exosomes and CNVs derived from PSCs can promote proliferation and migration of recipient cells and therefore can be used for the treatment of wound damage and skin aging. It has shown that exosomes derived from mouse embryonic stem cells can be used for cardiac regeneration in ischemic myocardium [106]. iPSC-EVs reduced hepatic stellate cell activation and hepatic fibrosis, which has been reported to reduce the tissue inhibitors of the fibrous markers a-smooth muscle actin, collagen Ia1, and fibronectin, and the tissue inhibitor of matrix metalloproteinases (MMP)-1 [107].

In addition, there have been therapeutic reports of exosomes derived from human iPSCs on tissue regeneration and aging of skin. Studies have shown that conditioned medium and exosomes derived from human iPSCs improve genotypic as well as phenotypic changes of human dermal fibroblasts (HDFs) induced by UV photoageing and natural senescence [108,109]. The conditioned medium and isolated exosomes from human iPSCs showed changes in cellular responses associated with skin aging; expression levels of MMP-1, MMP-3, and type I collagen, as well as proliferative and migratory phenotypes of HDFs. It is suggested that exosomes derived from PSCs contain functional factors that mediate intracellular delivery and rebalance the matrix in the aging skin by modulating the expression of aging-related genes and enhancing the content of structural proteins (i.e., collagen type I), thereby promoting the reconstitution of the dermal matrix in wound damaged and aged skin. It is suggested that exosomes derived from PSCs are similar to exosomes derived from MSCs, and contain various kinds of proteins, miRNAs, and mRNAs, DNAs, which may affect cellular physiologies. Branscome et al. compared iPSC-EV and MSC-EV [110]. As a result of protein analysis, iPSC-EV contains proteins involved in receptor tyrosine kinase signaling. MSC-EVs harbor proteins involved in pathways such as the Ras-related protein 1 (RASD1) pathway, and the Janus kinase-signal transducer and activator of transcription (JAK-STAT) pathway. iPSC-EV showed higher association with FGF-2, VEGF, and IL-4 than MSC-EV. In addition, when highly purified EVs derived from iPSCs were applied to senescent MSCs with elevated reactive oxygen species (ROS), the cellular ROS levels were reduced and aging phenotypes of senescent MSCs were alleviated [111]. It was notable that human iPSCs produced 16-fold more EVs than MSCs.

In recent years, new approaches in generating EV-mimetic nanovesicles, named cell-engineered nanovesicles (CNVs), have been introduced by serial extrusion of cells through membrane filters with diminishing pore sizes and thereby mechanically disrupting plasma membranes of cells [10,109,112] (Figure 2, lower panel). In a recent study, it was demonstrated that CNVs provide several advantages over conventional conditioned media-derived EVs respect to mass production [113]. When the total number of particles obtained as a relative to the same number of cells were compared, the average yield of the CENVs was over 70-fold higher than that of the conditioned media-derived EVs.

**Figure 3:**
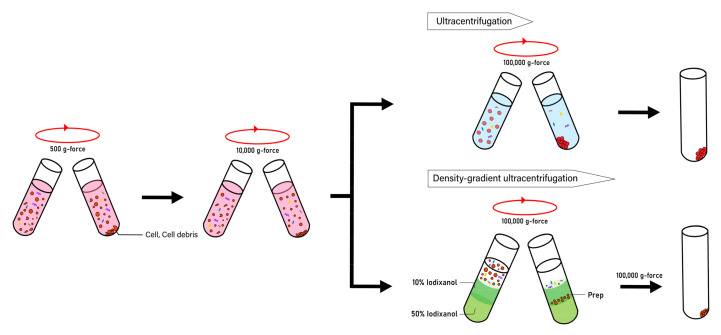
Purification of nano-sized vesicles. Nano-sized vesicles can be purified by precipitation, ultracentrifugation, or density-gradient ultracentrifugation methods. Prior to ultracentrifugation, cells and cell debris must be removed. Density gradient ultracentrifugation is superior to ultracentrifugation and precipitation methods in terms of purity.

Previously, it was shown that the ultracentrifugation method gives higher purity than the exosome isolation kit [114]. Although the ultracentrifugation method requires a high labor intensity, density gradient ultracentrifugation is superior to ultracentrifugation and precipitation methods in terms of purity (Figure 3). When the purities of EVs and CNVs were studied by observing the particle number-to-protein ratio, it was shown that CNVs have significantly higher purity than the EVs by more than 7-fold [113].

Previous reports have shown that CNVs derived from embryonic stem cells were able to self-renew bone marrow stem cells [115]. Recent studies have reported that CNVs derived from murine embryonic stem cell (mESC) enhanced the proliferative rate of murine skin fibroblasts by promoting expression levels of cell proliferation-related genes and proteins [116]. Comparative study showed that CNVs derived from iPSCs promoted proliferation and migration of HDFs in a similar manner to exosomes derived from iPSCs [113]. It also showed that CNVs down-regulated the expression of the senescence-associated marker SA-beta-gal, and cell cycle arrest and cellular senescence markers p53/p21. The results demonstrated that exosomes and CNVs derived from PSCs have therapeutic potential not only for proliferation, migration, and angiogenesis but also for treatment of wound damage, photoageing, and natural senescence (Table 3). These findings suggest that CENVs can be a useful alternative by overcoming the low productivity of naturally secreted exosomes.

## 6. Conclusions

**Figure 4:**
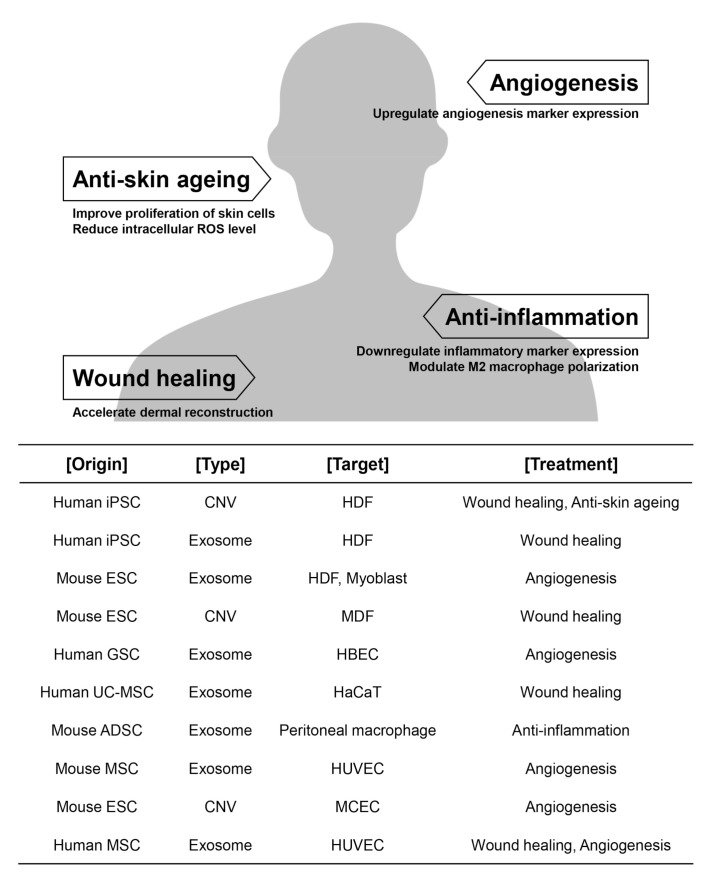
Therapeutic potential of nano-sized vesicles. Exosomes and CNVs derived from stem cells have therapeutic potential for not only proliferation, migration, and angiogenesis but also for treatment of wound damage, photoageing, and natural senescence. iPSC (induced pluripotent stem cell); ESC (embryonic stem cell); MSC (mesenchymal stem cell); GSC (glioma stem cell); UC-MSC (umbilical cord mesenchymal stem cell); ADSC (adipose-derived stem cell); CNV (cell-engineered nanovesicle); HDF (human dermal fibroblast); HUVEC (human umbilical vein endothelial cell); MDF (mouse dermal fibroblast); HBEC (human brain endothelial cell); HaCaT (human immortalized keratinocyte); MCEC (mouse cavernous endothelial cell).

Exosomes and EVs play important roles in intercellular communication between donor cells and recipient cells by the delivery of materials such as proteins and RNAs. Additionally, they are known to be associated with various normal and pathological conditions. The content and composition of the released exosomes is dependent on the state of the cells from which they originate. Exosomes are emerging as a desirable material for regenerative medicine due to their roles in anti-inflammation, the promotion of angiogenesis via proliferative and migratory phenotypes, as well as wound healing and anti-ageing properties (Figure 4). Although the conventional method (natural release mechanism) is generally used to obtain exosomes, new ways of generating exosome mimicking nanovesicles with higher yield are also being developed.

Here, we have summarized the biogenesis and biological functions of exosomes and CNVs in accordance with cell types, and their potential roles in therapeutic applications. The stem cell-derived exosome has the effect of inducing and promoting cell regeneration such as nerve cells and cardiomyocytes. For this reason, many studies are being conducted to apply it as a treatment strategy. The results have revealed the beneficial effects of exosomes and CNVs derived from cells, especially from PSCs on skin damaged by physical damage, photoageing, and on natural senescence. Exosomes and CNVs are advantageous in many ways, in terms of (1) stability, as they can stably preserve the components in lipid bilayer for long periods, (2) biocompatibility and delivery agent, as they have no cytotoxicity, natural cell targeting, and efficient delivery properties, (3) and therapeutic effects, as they have proliferative, migratory, and angiogenesis properties. Recent studies have shown that exosomes and CNVs can also be used in treating skin-related issues; ameliorating physiological deteriorations, and subsequent epigenetic alterations incurred by physical damage and natural senescence. Especially, many complicated processes are involved in skin aging and therefore further study is requisite to demonstrate the therapeutic potential of exosomes and CNVs. Recent reports suggest that exosomes and CNVs derived from PSCs could provide a technical advance in clinical applications and be used as a versatile tool for the treatment of wound damage and skin ageing.

## Figures and Tables

**Table 1:** Effect of cancer stem cell-derived extracellular vesicles (EVs) according to recipient cells or in vivo model.

EV Donor Stem Cells	EVs	EV Recipient Cells/In Vivo Model	Effect	Ref
Human glioma stem cells (U-251 cell line)	Exosomes	Endothelial cells (Human brain ECs)	Angiogenesis	[64]
Human liver cancer stem cells (Huh-7 human hepatocellular carcinoma cell line)	Exosomes	Endothelial cells (HUVEC)	Angiogenesis	[66]
Human renal cancer stem cells (5 Patients)	MVs	Endothelial cells (HUVEC)/lung premetastic niche in SCID mice In vivo	Angiogenesis	[65]
Human glioblastoma stem cell	Exosomes	Human monocytes	Induce M2 macrophages	[69]
Human clear cell renal cell carcinoma stem cell	Exosomes	Clear cell renal cell carcinoma in BALB/c nude mice In vivo	Promote epithelial-mesenchymal transition	[71]
Human thyroid cancer stem cell	Exosomes	Thyroid papillary carcinoma cell line (TPC-1)/Normal thyroid cell line (NTHY-ori-3)	Induce epithelial-mesenchymal transition	[72]

EV (extracellular vesicle); U-251 (human glioma stem cell); EC (endothelial cell); HUVEC (human umbilical vein endothelial cell); SCID (severe combined immunodeficiency); TPC (thyroid papillary carcinoma cell); NTHY (normal thyroid cell).

**Table 2:** Effect of multipotent stem cell-derived EVs according to recipient cells or in vivo model.

EV Donor Stem Cells	EVs	EV Recipient Cells/In Vivo Model	Effect	Ref
Human BM-MSCs	Exosomes	HUVEC, mouse C2C12 myoblast cell line/cardiotoxin muscle injury model in mice	Promote myogenesis and angiogenesis in vitro/Promote muscle regeneration in a muscle injury model	[88]
Human iPSC-MSCs	Exosomes	Injected systemically via the inferior vena cava in a rat model	Alleviate hepatic ischemia-reperfusion injury in rats (lowered Hepatocyte injury markers (AST and ALT) and inflammatory markers (TNF-α, IL-6, HMGB1))	[85]
Human iPSC-MSCs	Exosomes	Injected systemically into a murine ischemia/reperfusion injury model via the inferior vena cava	Induce primary hepatocytes and HL-7702 cells proliferation in vitro/Induce expression levels of proliferation markers (PCNA and PHH3) in vivo	[86]
Human iPSC-MSCs	Exosomes	Bone marrow MSCs derived from female ovariectomized rats (rBMSCs-OVX) in vitro/implanted into bone defects in ovariectomized rats	Repair bone defects via increased angiogenesis and osteogenesis in osteoporotic rats	[87]
Human iPSC-MSCs	Exosomes	HDFs and HUVECs/injected subcutaneously around wound sites in a rat model	Stimulate proliferation and migration/Accelerate re-epithelialization, facilitate angiogenesis and cutaneous wound healing in vivo	[95]
Human adiopose-MSCs	Exosomes	Primary HDFs/injected intravenously	Promote migration, proliferation, collagen synthesis/Accelerate cutaneous wound healing in vivo	[96]
Mouse BM-MSCs	EVs	Sciatic nerve crushed rat model	Promote peripheral nerve regeneration	[99]
Mouse UC-MSCs	EVs	Left sciatic nerves removed rat model	Promote peripheral nerve regeneration	[100]
Mouse BM-MSCs	EVs	SCI rat model	Improving the structural integrity of BSCB	[102]
Mouse BM-MSCs	EVs	Neuronal cells/SCI rat model	Anti-apoptosis of neuron cells	[103]
Mouse neural stem cell	EVs	Murine spinal neuron/rat model of SCI	Attenuate apoptosis and neuroinflammation	[104]
Human iPSC-MSCs	Exosomes	Human HaCaT keratinocytes and HDFs	Accelerate skin cell proliferation	[97]
Mouse MSCs	CNVs	Primary mouse skin fibroblasts/Injected IP into mouse skin wound model	Increase proliferation and migration in vitro/Accelerate healing in vivo	[98]

EV (extracellular vesicle); BM-MSC (bone marrow-derived mesenchymal stem cell); HUVEC (human umbilical vein endothelial cell); C2C12 (mouse myoblast cell); iPSC-MSC (induced pluripotent stem cell-derived mesenchymal stem cell); AST (aspartate aminotransferase); ALT (alanine aminotransferase); TNF-α (tumor necrosis factor-α); IL-6 (interleukin-6); HMGB1 (high mobility group box 1 protein); HL-7702 (normal human liver cell); PCNA (proliferating cell nuclear antigen); pHH3 (phosphohistone H3); rBMSC-OVX (bone marrow mesenchymal stem cell derived from female ovariectomized rat); HDF (human dermal fibroblast); UC-MSC (umbilical cord mesenchymal stem cell); SCI (spinal cord injury); HaCaT (human immortalized keratinocyte); CNV (cell-engineered nanovesicle); IP (intraperitoneal injection).

**Table 3:** Effect of pluripotent stem cell-derived EVs according to recipient cells or in vivo model.

EV Donor Stem Cells	EVs	EV Recipient Cells/In Vivo Model	Effect	Ref
Mouse ESCs	Exosomes	H9c2 myoblasts and HUVECs, cardiac progenitor cells (CPCs)	Enhance tube formation in vitro/Promote endogenous repair (augment neovascularization, myocyte proliferation, and survival) and enhance cardiac function in vivo	[106]
Murine ESCs	CNVs	Primary murine skin fibroblasts	Increase proliferation rate and growth factor secretion	[116]
Human iPSCs	Exosomes	HDFs	Stimulate proliferation, migration, and reduce photoaging, natural senescence markers (ameliorate skin ageing)	[109]
Human iPSCs	EVs	Senescent human MSCs	Improve proliferation, reduce intracellular reactive oxygen species (ROS) level (alleviate aging)	[111]
Mouse ESCs	CNVs	Murine MSCs	Enhance the proliferation rate	[115]
Human iPSCs	CNVs	Senescent human HDFs	Increase proliferation, migration, and reduce activity of senescence-associated genes	[113]
Human iPSCs	EVs	Hepatic stellate cell	Reduce hepatic stellate cell activation and liver fibrosis	[107]

EV (extracellular vesicle); ESC (embryonic stem cell); H9c2 (rat cardiomyoblasts); HUVEC (human umbilical vein endothelial cell); CPC (cardiac progenitor cell); CNV (cell-engineered nanovesicle); iPSC (induced pluripotent stem cell), HDF (human dermal fibroblast); MSC (mesenchymal stem cell).
